# Cathepsin Levels and Atrial Fibrillation Risk: Insights From Bidirectional and Multivariable Mendelian Randomization Analyses

**DOI:** 10.1155/ijog/8232758

**Published:** 2025-10-29

**Authors:** Fang Ye, Ruya Zhou, Haiying Lin, Liping Wu, Xianjun Wu

**Affiliations:** ^1^ Department of Cardiology, Lishui People′s Hospital, Lishui Hospital of Wenzhou Medical University, The First Affiliated Hospital of Lishui University, Lishui, Zhejiang, China; ^2^ Procurement Center, Lishui People’s Hospital, Lishui Hospital of Wenzhou Medical University, The First Affiliated Hospital of Lishui University, Lishui, Zhejiang, China; ^3^ Zijin Street Community Health Service Center, Lishui, Zhejiang, China

**Keywords:** atrial fibrillation, cardiovascular proteomics, cathepsin, genetic epidemiology, mendelian randomization

## Abstract

**Background:**

Atrial fibrillation (AF) is the most common cardiac arrhythmia, contributing to substantial morbidity, mortality, and healthcare burden. While genome‐wide association studies (GWAS) have identified numerous genetic variants linked to AF risk, the causal roles of proteolytic enzymes such as cathepsins remain poorly defined. This study employed bidirectional and multivariable Mendelian randomization (MR) approaches to investigate the causal relationship between genetically determined cathepsin levels and AF risk.

**Methods:**

Genetic instruments for nine cathepsins were derived from the INTERVAL study (*n* = 3301, European ancestry), using a significance threshold of *p* < 5 × 10^−6^ and stringent LD pruning (*r* ^2^ < 0.001, 10,000 kb window). Only SNPs with F‐statistics > 10 were retained. AF outcome data were obtained from a GWAS meta‐analysis comprising 60,620 cases and 970,216 controls of European descent. Two‐sample MR analyses were conducted using the inverse variance weighted (IVW) method, supported by MR‐Egger and weighted median approaches. Multivariable MR was used to adjust for correlated cathepsins, and reverse MR assessed bidirectional causality.

**Results:**

Genetically elevated cathepsin O levels were significantly associated with increased AF risk (IVW: *p* = 0.0025, OR = 1.06, 95% CI 1.02–1.10), and this association remained robust in multivariable MR (IVW: *p* = 0.0265, OR = 1.0571, 95% CI 1.0065–1.1102). A suggestive association for cathepsin B was observed only in multivariable MR (IVW: *p* = 0.0356, OR = 1.0279, 95% CI 1.0018–1.0547), but did not survive multiple testing correction. Sensitivity analyses supported the validity of these findings, and reverse MR showed no evidence of reverse causation.

**Conclusion:**

This study provides genetic evidence that elevated cathepsin O levels—and conditionally cathepsin B—are causally linked to increased AF risk. These findings highlight the potential role of proteolytic enzymes in AF pathogenesis and suggest novel therapeutic targets. All analyses were conducted in European‐ancestry populations; replication in diverse cohorts and mechanistic studies are warranted to validate and extend these insights.

## 1. Introduction

Atrial fibrillation (AF) is the most common cardiac arrhythmia encountered in clinical practice and is associated with increased morbidity, mortality, and substantial healthcare burden [1]. Despite advances in epidemiological and genetic research, the underlying mechanisms driving AF remain incompletely understood. Genetic predisposition plays a key role in AF pathogenesis, as evidenced by numerous risk loci identified through genome‐wide association studies (GWAS) [2, 3]. However, the biological pathways linking these genetic variants to AF development are still largely elusive.

Among the molecular candidates implicated in cardiovascular remodeling, cathepsins—a family of lysosomal proteolytic enzymes—have garnered attention for their roles in extracellular matrix turnover, inflammation, and tissue remodeling [4, 5]. While cathepsins have been studied in the context of atherosclerosis and heart failure, their potential involvement in AF has not been systematically evaluated. This represents a critical gap in our understanding of AF pathophysiology.

Mendelian randomization (MR) offers a powerful approach to assess causal relationships between biomarkers and disease outcomes by leveraging genetic variants as instrumental variables (IVs) [6]. Unlike observational studies, MR mitigates confounding and reverse causation, providing more robust evidence for causality.

In this study, we apply bidirectional and multivariable MR frameworks to investigate the causal impact of genetically predicted cathepsin levels on AF risk. Genetic instruments for nine cathepsins were derived from the INTERVAL study [7], and AF outcome data were obtained from the largest GWAS meta‐analysis to date [8]. By integrating these datasets, we aim to clarify whether specific cathepsins contribute to AF susceptibility and to explore their potential as therapeutic targets.

Our findings are expected to advance the mechanistic understanding of AF, refine genetic risk prediction, and inform future strategies for targeted prevention and management. This work contributes a novel dimension to AF research by linking proteolytic enzyme biology with arrhythmia risk through a rigorous genetic framework.

**Figure 1:**
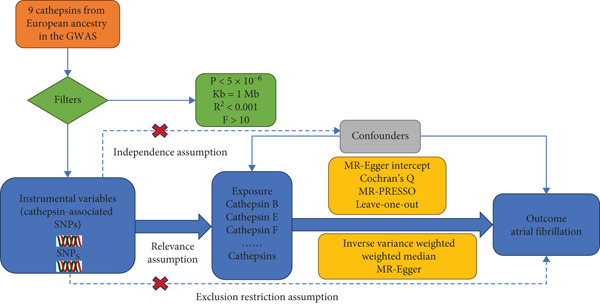
The three major hypotheses of two‐sample Mendelian randomization and the research flowchart illustrate the causal relationship between cathepsins and atrial fibrillation.

The conceptual framework of our MR approach—including the three core assumptions and analytical flow—is illustrated in Figure 1.

## 2. Methods

### 2.1. Data Acquisition

For the MR analysis investigating cathepsin levels, genetic instruments were sourced from the INTERVAL study, which included 3301 individuals of European descent [7]. All participants provided informed consent, and the study was approved by The National Research Ethics Service (approval number 11/EE/0538). Summary statistics are publicly available via the GWAS Central database (https://gwas.mrcieu.ac.uk).

AF outcome data were derived from a comprehensive meta‐analysis of GWAS, incorporating six independent cohorts—including deCODE, UK Biobank, and others—analyzing over 34 million genetic variants in 60,620 AF cases and 970,216 controls, all of European ancestry [8]. To minimize sample overlap and potential bias, exposure and outcome datasets were sourced from independent cohorts. While no known overlap exists, any residual overlap in two‐sample MR would be expected to bias estimates conservatively toward the null.

All analyses were conducted exclusively in European‐ancestry populations. While this enhances internal consistency, it may limit generalizability. Replication in non‐European cohorts is warranted to validate the observed associations.

### 2.2. Methodology and Instrumental Variable Selection

IVs were selected based on three core MR assumptions [9, 10]: (1) relevance—genetic variants must be robustly associated with the exposure (cathepsin levels); (2) independence—variants should not be associated with confounders; (3) exclusion restriction—variants must influence the outcome (AF) only through the exposure.

To satisfy these assumptions, we applied a stringent *p* value threshold of *p* < 5 × 10^−6^ to identify SNPs strongly associated with cathepsin levels. This threshold balances instrument strength and availability in proteomic GWAS, which often have modest sample sizes. All selected SNPs were pruned for linkage disequilibrium using an *r* ^2^ threshold of 0.001 and a clumping window of 10,000 kb [11].

Instrument strength was quantified using the F‐statistic, calculated as *F* = *R* ^2^(*N* − *K* − 1)/(*K*(1 − *R* ^2^)), where *R* ^2^ is the variance explained by the IVs, *N* is the sample size, and *K* is the number of IVs [12]. SNPs with F‐statistics < 10 were excluded to reduce weak instrument bias.

### 2.3. Analytical Strategy

The primary MR analysis was conducted using the inverse variance weighted (IVW) method, which applies the Wald ratio to each SNP and combines estimates via random‐effects meta‐analysis [13]. To ensure robustness, complementary methods including MR‐Egger [14] and weighted median [15] were employed.

Sensitivity analyses included Cochran’s Q test [16] for heterogeneity and the MR‐PRESSO suite [17] to detect and correct for horizontal pleiotropy and outlier effects. Leave‐one‐out analysis was performed to assess the influence of individual SNPs on overall estimates [18].

To account for correlated effects among cathepsins, multivariable MR was performed [19]. Reverse MR analyses were also conducted to evaluate bidirectional causality between AF and cathepsin levels using the same GWAS datasets.

Given the testing of nine cathepsins, multiple comparison correction was applied using Bonferroni (*α* = 0.0056) and false discovery rate (FDR) procedures. Adjusted *p* values are reported in Supplementary Table 1.

All statistical analyses were conducted in R (version 4.3.1), using the “TwoSampleMR” [20], “MR‐PRESSO” [17], and “MendelianRandomization” [19] packages. Causal estimates were expressed as odds ratios (ORs) with 95% confidence intervals (CIs), and statistical significance was defined as *p* < 0.05.

## 3. Results

### 3.1. Selection of Instrumental Variables (IVs)

Genetic variants for nine cathepsin enzymes were obtained from the INTERVAL study, while AF summary statistics were sourced from six independent GWAS cohorts. IVs were selected using a genome‐wide significance threshold of *p* < 5 × 10^−6^ and pruned for linkage disequilibrium (*r* ^2^ < 0.001 within a 10,000 kb window). All retained SNPs exhibited F‐statistics > 10, indicating strong instrument strength and minimizing weak instrument bias. Details of the selected SNPs are provided in Supplementary Table 2.

### 3.2. Primary Findings From MR Analysis

**Figure 2:**
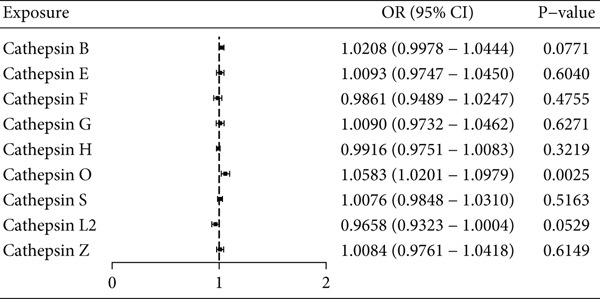
Univariate Mendelian randomization forest plot assessing the link between nine cathepsins and the likelihood of atrial fibrillation. The analysis was conducted using the inverse‐variance weighted approach to determine the potential causal connections among various cathepsins and the risk of atrial fibrillation.

In the univariable two‐sample MR analysis, genetically elevated levels of cathepsin O were significantly associated with increased AF risk (IVW: *p* = 0.0025, OR = 1.06, 95% CI 1.02–1.10). This association was consistently supported by complementary methods, including the weighted median approach (Table 1, Figure 2). No other cathepsins demonstrated significant associations with AF using the IVW method (Table 1, Figure 2).

**Table 1:** The results of univariable MR analysis between nine cathepsins and the risk of atrial fibrillation.

**Exposure**	**Outcome**	**Nsnp**	**MR-Egger**			**Weighted median**			**Inverse variance weighted**		
			***p* value**	**OR**	**95% CI**	***p* value**	**OR**	**95% CI**	***p* value**	**OR**	**95% CI**
Cathepisn B	Atrial fibrillation	20	0.756	1.008	0.957–1.063	0.051	1.031	0.999–1.064	0.077	1.021	0.998–1.044
Cathepisn E		11	0.236	1.052	0.973–1.137	0.443	1.019	0.972–1.068	0.604	1.009	0.975–1.045
Cathepisn F		12	0.557	0.968	0.872–1.075	0.969	1.000	0.957–1.046	0.475	0.986	0.949–1.025
Cathepisn G		12	0.833	0.990	0.904–1.084	0.612	1.013	0.965–1.063	0.627	1.009	0.973–1.046
Cathepisn H		11	0.725	0.996	0.973–1.019	0.397	0.992	0.973–1.011	0.322	0.992	0.975–1.008
Cathepisn L2		10	0.321	0.957	0.883–1.038	0.068	0.960	0.918–1.003	0.053	0.966	0.932–1.000
Cathepisn O		12	0.125	1.077	0.988–1.174	0.012	1.070	1.015–1.129	0.002	1.058	1.020–1.098
Cathepisn S		23	0.455	1.015	0.976–1.056	0.490	1.009	0.983–1.036	0.516	1.008	0.985–1.031
Cathepisn Z		13	0.120	0.966	0.927–1.006	0.151	1.027	0.990–1.064	0.615	1.008	0.976–1.042

*Note*: For univariable analyses across nine cathepsins, *p* values were adjusted using Bonferroni (*α* = 0.0056) and FDR (Benjamini–Hochberg). Cathepsin O remains significant after adjustment (see Supplementary Table 3). Multivariable *p* values are reported nominally; cathepsin B is suggestive and does not remain significant after multiplicity correction.

After applying multiple testing correction across the nine cathepsins, only cathepsin O remained statistically significant (Bonferroni‐adjusted *p* = 0.0225; FDR‐adjusted *p* = 0.0071), as shown in Supplementary Table 1.

**Figure 3:**
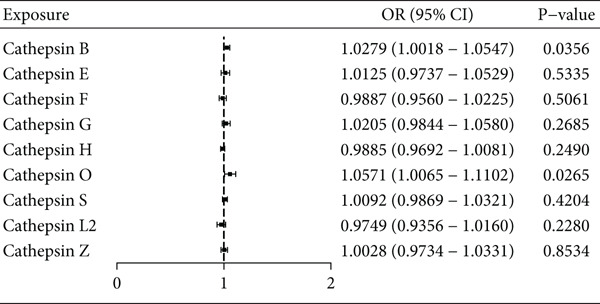
Multivariate Mendelian randomization forest plot using inverse variance weighting to explore the causal influence of nine cathepsins (cathepsins B, E, F, G, H, L2, O, S, and Z) on atrial fibrillation risk. This method was applied to ascertain the causal impact of these cathepsins on the incidence of atrial fibrillation.

In the multivariable MR framework, the association between cathepsin O and AF risk persisted after adjusting for correlated cathepsins (IVW: *p* = 0.0265, OR = 1.0571, 95% CI 1.0065–1.1102) (Figure 3). Additionally, a suggestive association was observed between cathepsin B and AF risk (IVW: *p* = 0.0356, OR = 1.0279, 95% CI 1.0018–1.0547); however, this did not survive multiple testing correction and should be interpreted with caution (Figure 3).

### 3.3. Sensitivity Analysis Outcomes

To assess the robustness of our findings and detect potential pleiotropy, we conducted sensitivity analyses using MR‐Egger and weighted median methods. Cochran’s *Q* test revealed no significant heterogeneity (*p* > 0.05), and the MR‐Egger intercept indicated no evidence of directional pleiotropy. These results are summarized in Supplementary Table 3.

Scatter plots and leave‐one‐out analyses (Supplementary Figures 1 and 2) further confirmed that no single SNP disproportionately influenced the overall estimates, supporting the stability of the causal associations.

### 3.4. Exploration of Reverse Causation

To evaluate the possibility of reverse causality, we performed reverse two‐sample MR analyses treating AF as the exposure and cathepsin levels as outcomes. No significant associations were observed, suggesting that AF does not causally influence circulating cathepsin levels. These results are presented in Supplementary Figure 3.

## 4. Discussion

This MR study provides novel insights into the causal relationship between circulating cathepsin levels and AF, a prevalent cardiac arrhythmia with substantial clinical burden. By leveraging genetic instruments from the INTERVAL study and robust AF GWAS data from six independent cohorts, we identified variants that satisfy the core MR assumptions—relevance, independence, and exclusion restriction—thereby enhancing the validity of our causal inferences.

Our primary findings demonstrate a statistically significant association between genetically elevated cathepsin O levels and increased AF risk. This association remained robust across multiple MR methods and persisted in a multivariable MR framework adjusting for other cathepsins. Importantly, cathepsin O was the only enzyme to retain significance after correction for multiple testing, underscoring its potential role as an independent and biologically relevant contributor to AF pathogenesis.

Cathepsin B exhibited a suggestive association with AF risk, emerging only in the multivariable MR analysis. However, this signal did not survive multiplicity correction and should be interpreted with caution. Its context‐dependent effect may reflect interactions within protease networks or inflammatory pathways, where cathepsin B modulates AF risk in the presence of other cathepsins.

Sensitivity analyses—including MR‐Egger, weighted median, Cochran’s *Q* test, and leave‐one‐out procedures—revealed no evidence of horizontal pleiotropy or heterogeneity, supporting the robustness of our findings. Reverse MR analyses further excluded the possibility of reverse causation, reinforcing the directional influence of cathepsin levels on AF risk.

Cathepsin O, as a cysteine protease, plays a pivotal role in the remodeling of the extracellular matrix (ECM), which is increasingly recognized as a precursor to AF. The proteolytic activity of cathepsin O facilitates the degradation of ECM components, which not only alters the structural integrity of the atrial tissue but also triggers the activation of profibrotic signaling pathways, particularly those mediated by transforming growth factor‐beta (TGF‐*β*). This activation leads to the phenoconversion of fibroblasts, resulting in an increased deposition of collagen and other ECM proteins, which further contributes to atrial fibrosis [21].

The resultant fibrotic environment creates a substrate that is conducive to the development of reentrant circuits, a hallmark of AF. Structural changes within the atrium, including dilatation and altered conduction properties, are intimately linked to these fibrotic processes [22]. Moreover, the interplay between cathepsin O activity and inflammatory mediators can exacerbate ECM remodeling, leading to a vicious cycle that perpetuates the substrate for AF [23–25].

Recent studies have suggested that targeting cathepsin O or its downstream signaling pathways may offer novel therapeutic avenues for preventing or mitigating AF. By inhibiting ECM degradation or modulating fibroblast activity, it may be possible to restore normal atrial structure and function, thereby reducing the incidence of AF episodes. Consequently, understanding the mechanistic contributions of cathepsin O to ECM remodeling and atrial fibrosis not only enhances our comprehension of AF pathogenesis but also highlights potential targets for intervention in this prevalent arrhythmia [26, 27].

Cathepsin B has garnered attention for its role in various inflammatory processes, which may be pivotal in the pathogenesis of AF. The modulation of NF‐*κ*B signaling by cathepsin B could lead to altered inflammatory cytokine profiles, thus impacting the electrical remodeling of the atria. This remodeling is characterized by changes in ion‐channel expression and function, which are critical for maintaining normal cardiac conduction [28–30].

Moreover, cathepsin B’s involvement in protease–protease interactions may further complicate the ion‐channel environment, potentially leading to conduction heterogeneity within the atrial myocardium. This heterogeneity is a well‐known substrate for AF, as it can create areas of re‐entry and disrupt normal electrical impulses. Understanding the precise mechanisms by which cathepsin B influences these pathways could unveil new therapeutic targets for preventing or treating AF, particularly in populations with heightened inflammatory responses. Further research is warranted to elucidate these interactions and their implications for AF risk stratification and management [31, 32].

The pathogenesis of AF is multifactorial, involving electrical, structural, and contractile remodeling of the atria [33]. Our findings suggest that cathepsins—particularly cathepsin O—may play a pivotal role in these processes, offering potential avenues for biomarker development and therapeutic intervention [5, 34]. The conditional signal for cathepsin B invites further investigation into the coordinated regulation of proteolytic enzymes and their collective impact on cardiac function [35].

Future research should aim to replicate these findings in more ethnically diverse populations and explore the biological mechanisms underlying the association between cathepsins and AF. Experimental studies are needed to confirm the causal roles of cathepsins in AF and to understand the directionality and magnitude of their effects. Furthermore, the development of cathepsin‐targeted therapies could be explored as a novel strategy for AF prevention and management.

One of the strengths of this study lies in its comprehensive analytical framework, which included univariable and multivariable MR approaches, rigorous sensitivity analyses, and bidirectional testing. This design enabled us to assess causality with confidence and to explore the potential interplay among cathepsins. The absence of reverse causality strengthens the plausibility of a unidirectional effect from cathepsin elevation to AF risk.

Nonetheless, several limitations warrant consideration. All data were derived from European‐ancestry populations, which may limit the generalizability of our findings to other ethnic groups. Future studies should aim to replicate these results in diverse populations to assess the consistency and transferability of the observed associations. Additionally, while MR reduces confounding and reverse causation, it relies on key assumptions that cannot be empirically verified in full. Residual pleiotropy or unmeasured confounding may still influence the estimates.

## 5. Conclusions

In conclusion, our study provides genetic evidence supporting a causal role for cathepsin O—and suggestively for cathepsin B—in AF risk. These findings enhance our understanding of the molecular mechanisms underlying AF and highlight proteolytic enzymes as potential therapeutic targets. Further experimental and translational research is needed to validate these associations and to explore cathepsin‐targeted strategies for AF prevention and management.

## Ethics Statement

Public summary data were used and therefore no ethics approval was required.

## Conflicts of Interest

The authors declare no conflicts of interest.

## Author Contributions

Xianjun Wu designed the study and contributed to the analysis and interpretation of data. Fang Ye, Ruya Zhou, Haiying Lin, and Liping Wu did the statistical analysis and prepared the tables and figures. Fang Ye, Ruya Zhou, and Xianjun Wu wrote the first draft of the manuscript. Haiying Lin provided further data interpretation. All authors contributed to drafting the work or revising it critically for important intellectual content and made substantial contributions to the concept and design of the study and acquisition, analysis, and interpretation of data.

## Funding

This work was supported by the Zhejiang Provincial Medical Association Clinical Medicine Special Fund Project (2023ZYC‐Z49).

## Supporting Information

Additional supporting information can be found online in the Supporting Information section.

## Supporting information


**Supporting Information 1** Supplementary Table 1: Adjusted *p* values for univariable MR analyses of cathepsins and AF risk (IVW method).

**Supporting Information 2** Supplementary Table 2: Characteristics of single nucleotide polymorphisms (SNPs) serving as instrumental variables (IVs).

**Supporting Information 3** Supplementary Table 3: Pleiotropy and heterogeneity analyses of forward MR between cathepsins and atrial fibrillation.

**Supporting Information 4** Supplementary Figure 1: Scatter plot of SNPs associated with cathepsin O and the risk of atrial fibrillation. The plot related the effect sizes of the SNP–cathepsin O association (*x*‐axis) and the SNP − Atrial fibrillation associations (*y*‐axis) with 95% confidence intervals. The regression slopes of the lines correspond to causal estimates using five Mendelian randomization methods.

**Supporting Information 5** Supplementary Figure 2: Leave‐one‐out analysis of Mendelian randomization (MR) estimates of genetic risk of cathepsin O on Atrial fibrillation. Black boxes corresponding to each of the single nucleotide polymorphisms (SNPs) denote odds ratios (OR) derived from inverse variance weighted (IVW) after leaving the corresponding SNP in turns. The red box corresponding to “ALL” indicates the pooled IVW MR estimate. Horizontal lines denote a 95% confidence interval (CI).

**Supporting Information 6** Supplementary Figure 3: The results of reverse MR analysis between the risk of atrial fibrillation and various cathepsins.

## Data Availability

The datasets analyzed in this study are publicly available. Genetic instruments for cathepsins were obtained from the INTERVAL study via GWAS Central (https://gwas.mrcieu.ac.uk), and atrial fibrillation outcome data were sourced from a published GWAS meta‐analysis. For further inquiries, please contact the corresponding author.
