# Supplementary figures and images for: Cathepsin Levels and Atrial Fibrillation Risk: Insights From Bidirectional and Multivariable Mendelian Randomization Analyses
Source: Int J Genomics. 2025 Oct 29;2025:8232758. doi: 10.1155/ijog/8232758 (PMC12569522; doi:10.1155/ijog/8232758)

# MR Test

- Inverse variance weighted
- MR Egger
- Simple mode
- Weighted median
- Weighted mode

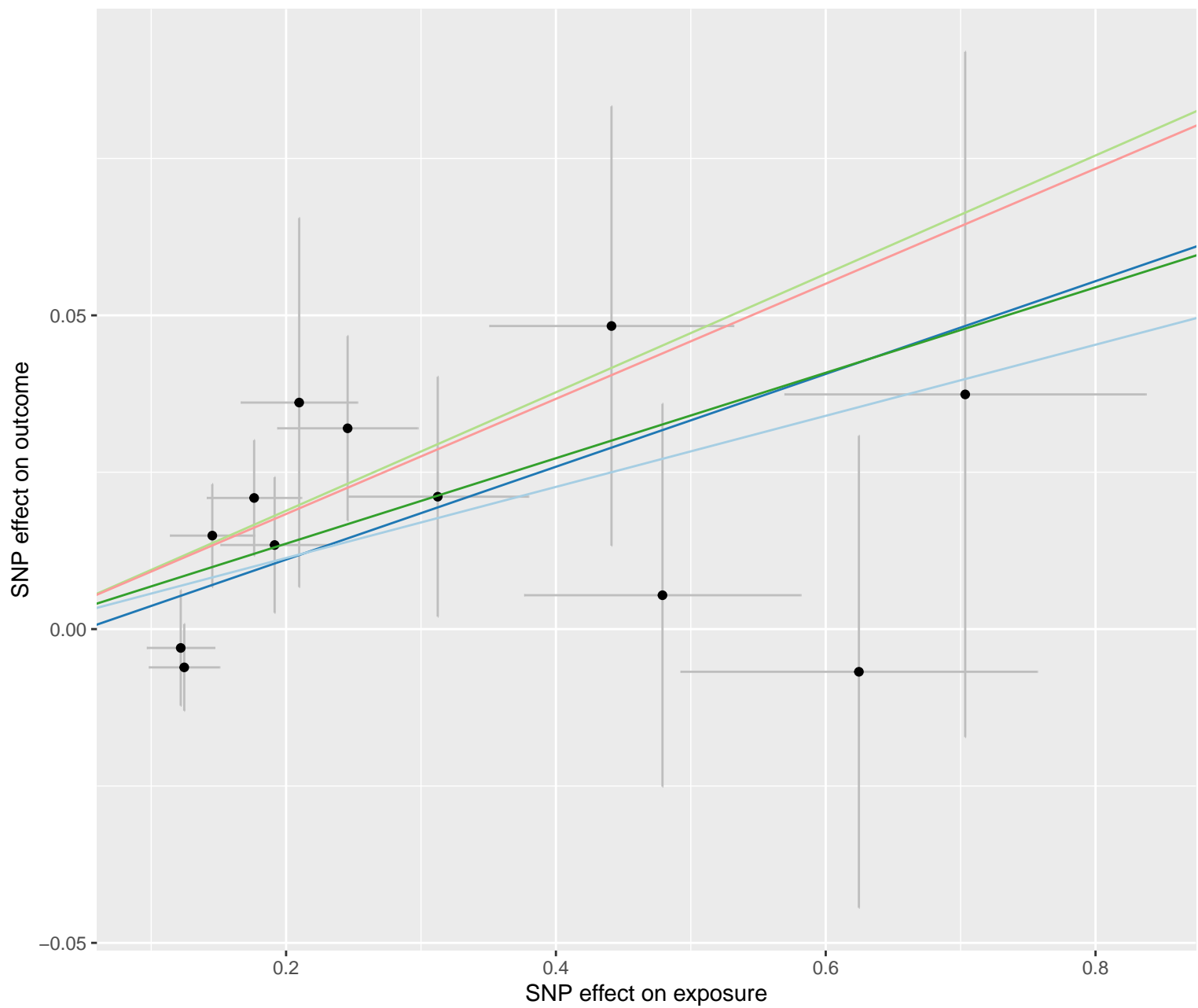

Supplement: Supplementary file 4 — Supporting Information 4 Supplementary Figure 1: Scatter plot of SNPs associated with cathepsin O and the risk of atrial fibrillation. The plot related the effect sizes of the SNP–cathepsin O association (x‐axis) and the SNP − Atrial fibrillation associations (y‐axis) with 95% confidence intervals. The regression slopes of the lines correspond to causal estimates using five Mendelian randomization methods. [file IJOG-2025-8232758-s005.pdf]

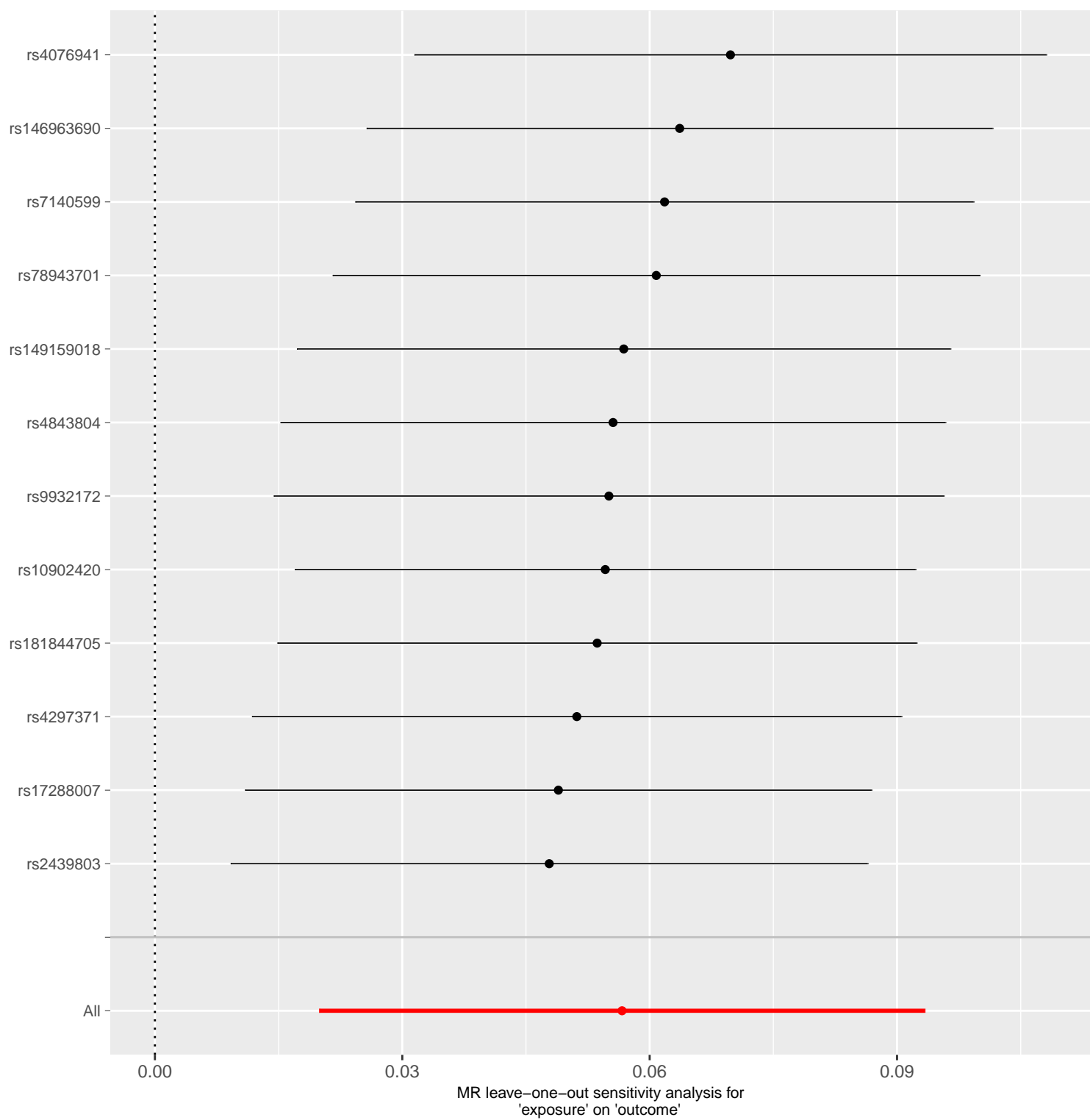

Supplement: Supplementary file 5 — Supporting Information 5 Supplementary Figure 2: Leave‐one‐out analysis of Mendelian randomization (MR) estimates of genetic risk of cathepsin O on Atrial fibrillation. Black boxes corresponding to each of the single nucleotide polymorphisms (SNPs) denote odds ratios (OR) derived from inverse variance weighted (IVW) after leaving the corresponding SNP in turns. The red box corresponding to “ALL” indicates the pooled IVW MR estimate. Horizontal lines denote a 95% confidence interval (CI). [file IJOG-2025-8232758-s002.pdf]
