# Supplementary material for: Cathepsin Levels and Atrial Fibrillation Risk: Insights From Bidirectional and Multivariable Mendelian Randomization Analyses
Source: Int J Genomics. 2025 Oct 29;2025:8232758. doi: 10.1155/ijog/8232758 (PMC12569522; doi:10.1155/ijog/8232758)

Outcome	method	nsnp	pval	OR(95%CI)
Cathepsin O	Inverse variance weighted	109	0.0962	1.07 (0.99−1.15)
Cathepsin B	Inverse variance weighted	109	0.2198	1.06 (0.97−1.16)
Cathepsin S	Inverse variance weighted	109	0.3326	1.04 (0.96−1.13)
Cathepsin F	Inverse variance weighted	109	0.5794	1.02 (0.94−1.12)
Cathepsin Z	Inverse variance weighted	109	0.6721	1.02 (0.94−1.10)
Cathepsin L2	Inverse variance weighted	109	0.9510	1.00 (0.93−1.08)
Cathepsin G	Inverse variance weighted	109	0.9999	1.00 (0.93−1.08)
Cathepsin E	Inverse variance weighted	109	0.8789	0.99 (0.92−1.07)
Cathepsin H	Inverse variance weighted	109	0.5878	0.98 (0.91−1.06)

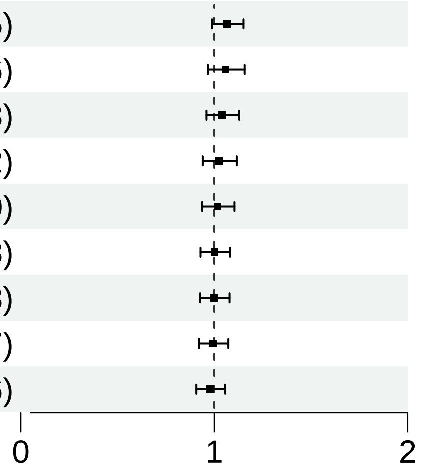

Supplement: Supplementary file 6 — Supporting Information 6 Supplementary Figure 3: The results of reverse MR analysis between the risk of atrial fibrillation and various cathepsins. [file IJOG-2025-8232758-s004.pdf]
